# The Irish Trainee Emergency Research Network (ITERN): five years of collaboration

**DOI:** 10.1007/s11845-023-03499-z

**Published:** 2023-08-16

**Authors:** James Foley, Jeffrey Mulcaire, Marcus Jee, Andrew Patton, Etimbuk Umana

**Affiliations:** 1 Irish Trainee Emergency Research Network (ITERN), Dublin, Ireland; 2 Emergency Department, North Bristol Trust NHS, Bristol, UK; 3 Emergency Department, Cork University Hospital, Cork, Ireland; 4 Emergency Department, Galway University Hospital, Galway, Ireland; 5 Emergency Department, St. Vincent’s University Hospital, Dublin 4, Ireland; 6 Emergency Department, Mater Misericordiae Hospiztal, Dublin 7, Ireland

**Keywords:** Collaboration, Emergency medicine, Research, Research network, Trainees

## Abstract

In 2018, a group of Irish emergency medicine (EM) trainees recognised their common interest in collaborative research and the difficulties that trainees can encounter when trying to broaden their research capacity, prompting the beginning of the Irish Trainee Emergency Research Network (ITERN) journey. Trainee-led collaboratives have been shown to be feasible and have the potential to deliver impactful research projects, generating an evidence base that may not have been possible without collaboration. This article describes the successes and achievement of ITERN and describes the processes and challenges that a trainee-led research network can encounter. The authors believe that trainee-led collaboratives can deliver powerful and impactful research for patients and broaden the research capacity of individuals, hospitals, and groups of healthcare professionals.

## Introduction

“By trainees, for trainees”

This is one of the core principles that formed the basis for the establishment of the Irish Trainee Emergency Research Network (ITERN). In 2018, a group of Irish emergency medicine (EM) trainees recognised their common interest in collaborative research and the difficulties that trainees could encounter when trying to broaden their research capacity, leading to the inception of the ITERN journey. Trainee-led collaboratives have been shown to be feasible and have the potential to deliver impactful research projects, generating an evidence base that may not have been possible without collaboration [1]. Examples such as the Irish Research Surgical Collaborative, West Midlands Research Collaborative, Welsh Geriatric Registrar-Led Research Network, and Trainee Emergency Research Network (TERN) have shown their benefits in recent years with the development of priority documents, research production, and evidence translation [1–5]. The ITERN research strategy has been published previously, and since its inauguration, the depth and breadth of the research output from the network provides a proof of concept of the vision of a trainee-led EM research collaboration [6].

## Research activity and processes

**Fig. 1:**
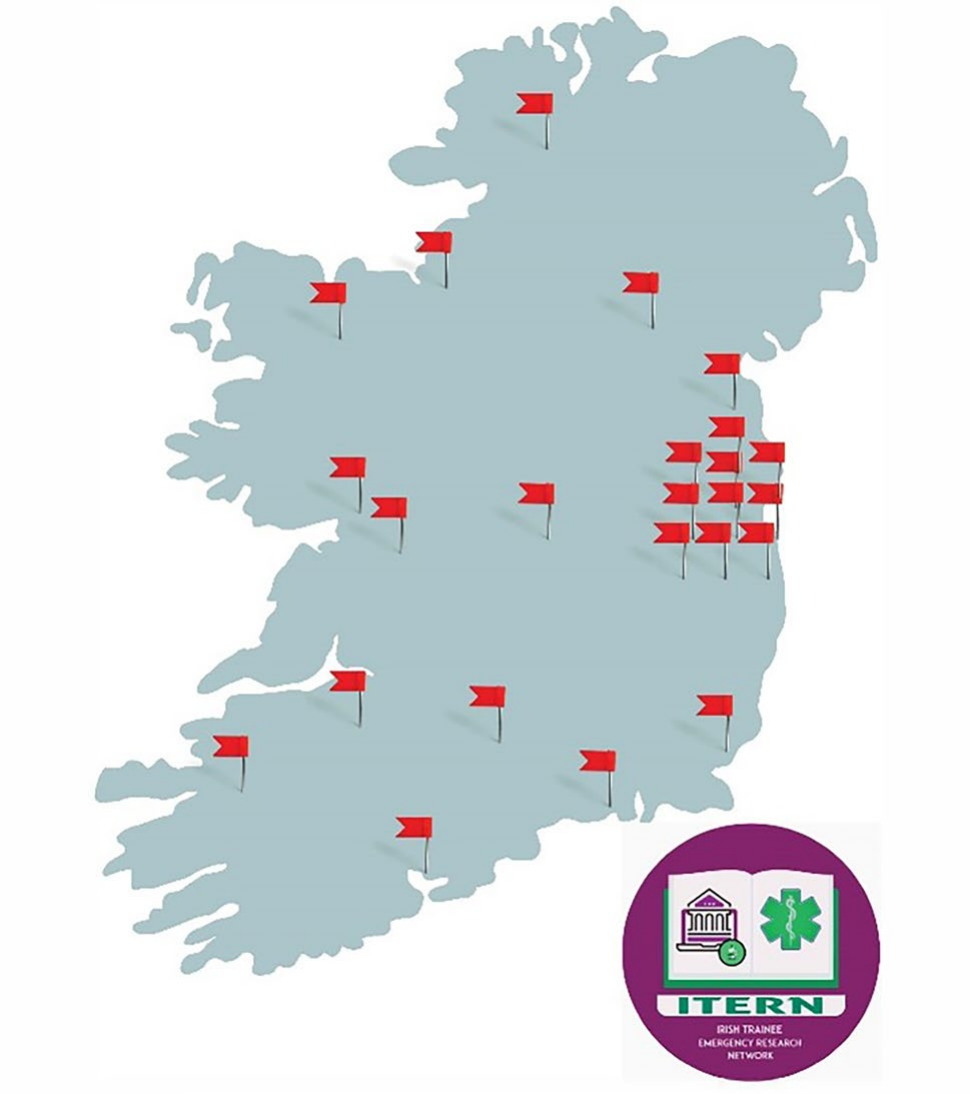
ITERN ED involvement in 2018–2023

At the time of writing, ITERN is currently recruiting site leads to their fifth research project. Three studies have been run independently [National Emergency Research Airway Audit (NERAA) [7], Emergency Department End of Life Care (EDEL), and Chest Drains in Emergency Department Audit (CHEDA)], whilst two projects were in collaboration with TERN [COVID-19 Emergency Response Assessment (CERA) [8, 9] and Acute Coronary Syndrome in the ED (ACS:ED)]. These studies have engaged a multitude of EM trainees in collaborative research, with 23 sites enrolling in the EDEL study for example. This has provided trainees with opportunities to run a research project in their local emergency departments (EDs), engage with local ethics committees, deliver the project autonomously under the supportive structure of ITERN, and developing the capacity of EM research in the future. Broadening the network not only enhances the opportunities for trainees, but also enhances the research project, converting it from a possible local study into a multi-site project, delivering more powerful data with potential vectors for change. The EDs in Ireland that ITERN have collaborated with are shown in Fig. 1.

Collaboration is a key component of network development. TERN and ITERN have been in partnership since the development of both networks, collaborating on projects and broadening the research capacity of EM in the UK and Ireland. The impact of this was shown in the CERA study which obtained over 5000 survey responses and has served as the sentinel study analysing the psychological impact of a pandemic on frontline doctors, subsequently contributing to policy recommendations to improve staff recruitment and retention [10, 11]. Additionally, whilst the ITERN model is trainee-led and delivered, the Irish Association of Emergency Medicine (IAEM) Academic Research Committee has been forthcoming with support and guidance, recognising the benefits of trainee-led research collaborations. Indeed, two of the ITERN projects (NERAA and CHEDA) were beneficiaries of the IAEM annual research bursary. This further demonstrates support from consultants in EM with a research interest in multi-site collaborations. The Royal College of Surgeons in Ireland (RCSI) has also collaborated with ITERN in recent years, with the Research and Innovation Department providing use of Research Electronic Data Capture (REDCap™) for projects, allowing an encrypted and user-friendly database for data entry. This relationship is a key component of the success of ITERN to date and cements the importance of collaborations when attempting to perform large-scale research projects.

## Challenges

At the outset, there was uncertainty around the success of ITERN. The enthusiasm and engagement of trainees nationally have been warmly welcomed. These have significantly driven the network forward to where it is today; however, during that time, there have been challenges to overcome. Currently, to obtain multi-site ethical approval in Ireland, this is possible through the National Office for Research Ethics Committees, but specifically related to clinical trials of investigational medicinal products or for clinical investigations of medical devices. Recently, the office also established a committee to deliver an expedited process for review COVID-19-related health research. However, outside of these research areas, obtaining ethical approval nationally requires that investigators submit their projects to each individual research and ethics committee (REC) in Ireland, of which there are approximately 32 committees in existence [12]. Whilst all ITERN studies have ethical approval in at least one site prior to dissemination nationally, the variability of approaches of the RECs could impact on the perception of the studies. Feedback can vary from protocol amendments, alternative patient information leaflets to rebranding of study documentation, or even study rejection (despite the study being approved elsewhere in Ireland). This process is time consuming and therefore, can delay project commencement on one site, despite the approval of others. Multi-site ethical approval is not insurmountable however, and the success of the EDEL study at 23 sites shows this. It is noteworthy that EDEL was a low-risk study that did not involve patients, making ethical approval a less challenging process. Therefore, ITERN welcomes the Health Service Executive (HSE) plans to reform the ethical approval process, providing more access to research for a wider population and a more consistent approach to approving research projects nationally [12].

## Data protection

Data protection laws have come into place in the last 5 years in Ireland and heralded changes to legislation and conduct of research. This created challenges around conducting multi-site research as transfer of data to a central hub is necessary to undertake analysis. Ensuring with data regulation, using enhanced software like REDCap™ has further helped in this arena. Also, training in the general data protection regulation for all ITERN members is paramount as well as collaborating with data protection officers (DPOs) within each individual site. ITERN has been committed to overcoming these challenges and moving this trainee-led organisation to next frontier in relation to data protection.

## The future

Overwhelmingly, the feedback from trainees in Ireland has been positive, and a research collaborative such as ITERN provides opportunities that may not have existed without such a venture. Encouraging a research-positive culture, both locally and nationally, is important not only for trainees but also for patients with wider access to research, with the aim of improving care and treatment options. It is well established that hospitals engaged in research have better mortality rates and that patients who attend research-active hospitals are better informed about their health [13–15]. It is therefore important for individuals, specialty groups, and hospitals to develop, promote, and provide a sustainable research environment. ITERN aims to provide such a platform for EM trainees, thereby broadening the research capacity of EM nationally. To date, projects have included observational or survey data. To progress the network in the future, a clinical trial would be the next logical step. ITERN therefore welcomes the development of the HSE National Policy for Consent in Health and Social Care Research, and in particular, Sect. 6 regarding the deferral of consent in emergency situations [16]. Given the acuteness of the issues patients present to EDs with, it can be difficult to involve them in research due to the time-critical nature of their treatments. This document will allow more patients to be involved in research and give clinicians the autonomy to ethically include patients in trials and consent them at a more appropriate time. For ITERN, this is a highly positive development which will likely be applicable for future emergency research collaborative projects.

## Conclusion

ITERN was a vision among a small group of trainees and has built itself up into a viable trainee-led research network over the last 5 years. By providing a platform for EM trainees to engage in research and support it through a sustainable model, the network has grown, resulting in impactful and powerful research outputs. What will the future hold? It is anticipated that new trainees will become involved as leaders within the research network. Also, the types of research will diversify into more extensive quantitative and qualitative studies. Importantly, patients will ultimately benefit from these ventures. ITERN has been successful to date, and the authors hope that it will continue to develop and form an integral part of the research strategy for Irish EM.
